# Effects of LED supplemental lighting on the growth and metabolomic profile of *Taxus baccata* cultivated in a smart greenhouse

**DOI:** 10.1371/journal.pone.0266777

**Published:** 2022-07-08

**Authors:** Ilaria Chiocchio, Alberto Barbaresi, Lorenzo Barbanti, Manuela Mandrone, Ferruccio Poli, Daniele Torreggiani, Mattia Trenta, Patrizia Tassinari

**Affiliations:** 1 Department of Pharmacy and Biotechnology, University of Bologna, Bologna, Italy; 2 Department of Agricultural and Food Sciences, University of Bologna, Bologna, Italy; Indian Council of Agricultural Research-Directorate of Mushroom Research, INDIA

## Abstract

Light emitting diode (LED) lamps are increasingly being studied in cultivation of horticultural, ornamental and medicinal plants as means to increase yield, quality, stress resistance, and bioactive compounds content. Enhancing the production of metabolites for medicinal or pharmaceutical use by regulating LED intensity and spectra is a challenging subject, where promising results have been achieved. Nevertheless, some species have been poorly investigated, despite their interest as a source of medicinally active substances, with particular reference to LED effects at the plant cultivation level. This study evaluates the effects of supplementary top-light LED treatments on *Taxus baccata*, one of the main sources of taxane precursors. Blue, red and mixed red–and-blue spectra were tested at 100 μM m^-2^ s^-1^. Moreover, 50 and 150 μM m^-2^ s^-1^ intensities were tested for the mixed spectrum. All treatments were set for 14 hours a day and were tested against natural light as control treatment, in a controlled environment, from 19 August to 9 December 2019, this latter date representing 112 days after treatment (DAT) began. A smart monitoring and control system powered by environmental and proximal sensors was implemented to assure homogeneity of temperature, humidity, and base natural light for all the treatments. It resulted in negligible deviations from expected values and reliable exclusion of confusing factors. Biometric measurements and ^1^H-NMR based metabolomic analysis were performed to investigate growth and phytochemical profile throughout the trial. One-way ANOVA showed that supplemental LED lighting increased plant height and number of sprouts. Considering the mixed red–and-blue spectrum, plant height increased almost proportionally from control to 100 μM m^-2^ s^-1^ (+20% at 112 DAT), with no further increase at higher intensity. The number of sprouts was strongly enhanced by LED treatments only in the early phase (48.9 vs. 7.5 sprouts in the averaged 50, 100 and 150 μM m^-2^ s^-1^ vs. the control at 28 DAT), with no differences related to intensity in the very early stage, and more persisting effects (up to 56 DAT) for higher intensities. After the very early growth stages (28 DAT), plant vigor showed a modest although significant increase over time compared to the control, with no differences related to light intensity (0.81 vs. 0.74 of NDVI in the averaged 50, 100 and 150 μM m^-2^ s^-1^ vs. the control, across 56, 84 and 112 DAT). The different spectra tested at 100 μM m^-2^ s^-1^ showed no significant differences in growth parameters, except for a slight beneficial influence of blue (alone or with red) compared to only red for sprouting. According to the metabolomic analysis, treated plants at 28 DAT were characterized by the highest content of sucrose and aromatic compounds. Signals of a putative taxane were detected in the ^1^H NMR profiles of plants, which were compared to the spectrum of baccatin III standard. However, the intensity of these spectral signals was not affected by the treatment, while they increased only slightly during time. Light at 150 μM m^-2^ s^-1^ induced the strongest variation in the metabolome. Conversely, light composition did not induce significant differences in the metabolome.

## 1. Introduction

LEDs for indoor cultivation mainly emit useful radiation, namely radiation able to enhance productivity by acting on the morphological and metabolic responses of plants. Since the maximum absorption of light by photoreceptors occurs in two wavelength ranges of the electromagnetic spectrum (650–665 nm and 450–470 nm, corresponding to the red and blue regions of the visible spectrum), the useful radiation for plants coincides with the wavelength range between 400 and 700 nm (PAR, photosynthetically active radiation). Photoreceptors sensitive to blue light (cryptochromes) and red light (phytochromes) stimulate transduction signals that act on plant morphology and physiology via different mechanisms of action [1, 2]. Red light appears to be related to a stimulus to flowering in long-day species [3], and internode elongation [4], while blue light appears to be related to plant phototropism [5] and stomata opening [6].

Given these premises, and due to their physical characteristics (narrow spectral bands, non-thermal photon emission, longer life, energy saving), LED lamps with a combined red and blue spectrum are currently the most widely used light source to optimize growth and metabolism of plants in a controlled environment [7–9]. The physical properties of LEDs make them suitable for use both in intra-canopy (aka, inter-lighting) and top-lighting systems [10], and both in horticulture and ornamental plants production, allowing reductions in energy consumption while maintaining optimal photon flux values of the incident radiation; for this reason, they are a valid source of light energy in greenhouse environments, especially during the winter season, exerting positive effects on crop quality, disease resistance, and bioactive compounds content [9]. Supplemental LED inter-lighting, relevant in particular for high-wire vegetable cultivations, has proven to increase yield and improve fruit quality in various all-year round greenhouse crops [11]. Supplemental top-lighting has proved to be beneficial in improving plant growth, morphology and quality of ornamental greenhouse-grown plants, with different results depending on spectral composition [12].

The applications of LED lighting to the cultivation of plants for both horticultural and medicinal use are numerous, and their effectiveness is widely demonstrated. Exposure to spectra of variable composition (blue, red and white light) showed effects on the organoleptic characteristics (color, aroma and flavor) in chili pepper cultivars (*Capsicum annuum* L.) [13], and on yield and characteristics (such as crispness, sweetness, shape, color, and accumulation of chlorophylls, carotenoids, soluble proteins and sugars, and nitrates) in different species of leaf crops (*Lactuca sativa* L., *Spinacia oleracea* L., also in hydroponic cultivation), and aromatic and medicinal plants (*Mentha arvensis* L., *Glycyrrhiza uralensis* Fisch. ex DC.) [14, 15]. Blue light, in particular, showed effects on production of metabolites with nutritional properties (carotenoids and other pigments, glycosinolates, minerals) [16]. Exposure to appropriate LED spectra can also trigger the synthesis of antioxidants and bioactive compounds, which in turn contribute to the improvement of the nutritional properties of the species used in horticulture; it can directly increase nutrient content, reduce microbial contamination, induce systemic resistance to fungal pathogens, affect the post-harvest ripening times of fruit and vegetables [9].

Besides the huge research effort focused on the use of LEDs to promote indoor/hydroponic cultivation of edible and ornamental plants, an increasing attention has been paid to the study of LEDs in medicinal and aromatic plants. It is acknowledged that medicinal and aromatic plants are influenced by micro-climatic conditions, namely light. This includes not only plant growth and development, but also secondary metabolite production [17]. However, the way and degree in which light intensity, duration and spectral composition influence plant behavior and the yield of secondary metabolites vary from species to species, and no univocal trend may be outlined. LED technology has been used to stimulate plant growth and production of metabolites for medicinal or pharmaceutical use in numerous species. Monochromatic light or spectral combinations of more wavelengths were used, either in substitution of or in addition to natural light, both on crops in controlled environments and on tissue cultures.

Fukuyama et al. [18–20] have shown that there are optimal frequencies and intensities of LED light (both red monochromatic and combined red-blue-UVA) capable of stimulating, increasing and optimizing the production of the monoterpenoid alkaloids vindoline and catharanthin, precursors of anticancer vinblastine and vincristine, in plants and leaf tissue of *Catharanthus roseus* (L.) G. Don. In the same species, Molchan et al. [21] determined the optimal PPFD (Photosynthetic Photon Flux Density) values for increase in dry weight and for alkaloid synthesis.

Supplemental night lighting with blue LEDs (440–540 nm, peak at 469 nm) showed positive effects both on main growth parameters and production of secondary metabolites of pharmacological interest (mainly flavonoids and polyphenols with antitumor activity) in plants of *Anoectochilus roxburghii* (Wall.) Lindl. grown in greenhouses [22].

In cultures of root apexes of *Artemisia annua* L. exposed to red light (660 nm), higher biomass values and a higher artemisinin content were measured compared to crops exposed to white light [23]. In cell cultures of *Artemisia absinthium* L., Tariq et al. [24] demonstrated that white light treatment, compared to other spectra, stimulated both a higher frequency of growth and higher total phenol and flavonoid values.

There are studies on the effects of treatment with LED spectra on conifers, focusing on growth parameters, on the physiology of gas exchange and on the induction of gene expression in seedlings of species of forest interest (*Picea abies* (L.) H. Karst. and *Pinus sylvestris* L.) [25, 26]. Despite the great interest in the *Taxus* genus as a source of substances with antitumor activity, studies on the effects of LEDs for medicinal purposes in *Taxus* spp. are still in the initial phase.

Su et al. [27] report a study with light spectra obtained through colored filters on *Taxus yunnanensis* WCCheng & LKFu (synonymous with *Taxus wallichiana* Zucc. var. *wallichiana*): the blue and red filters in different combinations confirmed positive effects on growth, physiology and synthesis of metabolites of pharmacological interest. Additionally, experiments using LEDs have largely been performed on plant tissue cultures, which, nevertheless, may lead to results not consistent with those obtained on whole plants.

In the latest years, the agri-food sector has been showing an increasing interest in smart monitoring and modelling systems powered by ICT (Information and Communications Technology) and IoT (Internet of Things), since their application has set the premise for an efficient, sustainable and traceable management of agricultural systems, with positive impact on the market also due to the risen awareness of the consumers. Applications concerning the monitoring of field and indoor environmental conditions (for plant growing, livestock raising and food processing structures) represent a crucial part of those systems [28, 29]. Moreover, spectral vegetation indices based on remote and proximal sensing have been widely used to estimate and analyze the vigor and phenology of plants, their reaction to stress conditions, degree in vegetation greenness, and variation of canopy chlorophyll content [30, 31]. Among them, the normalized difference vegetation index (NDVI) [32] is the vegetation index most widely used and one of the most reliable [33, 34]. Recently, specific developments proposed solutions to check and monitor several metrics from crop and animal conditions to food quality. Moreover, the possibility to send, receive and store data in real time and the potentialities of big data analysis, allow the precision and efficacy of the monitoring to be enhanced [35–38]. These systems also determine a dramatic improvement in the accuracy of plant experiments in controlled environments (such as greenhouses), based on the analysis of the spatial and temporal variability/homogeneity of environmental conditions affecting crop development, growth, quantitative and qualitative characteristics. These systems provide control of the designed conditions, record of the variable trends, identification and prevention of undesired conditions that could endanger or bias the trial. Moreover, monitored data could be helpful to define additional findings in research [39]. In particular, in studies aimed to assess the effect of one variable only, the homogeneity of other variables across all the treatments is a fundamental requirement.

European yew, *Taxus baccata* L., is considered one of the main sources of taxane precursors currently usable in the pharmacological field.

The poor knowledge about the potential effects of targeted lighting on this species, and the new opportunities disclosed by smart monitoring systems in the agricultural sector, constitute the premise for evaluating the effects of supplementary LED treatments on *Taxus baccata* growth and metabolomic profile.

Plants were grown in pots in a smart greenhouse, in order to maintain proper environmental conditions and ensure homogeneity among all the treatments, and^1^H NMR based metabolomic analysis was performed to investigate *T*. *baccata* metabolome and its response under different LED light treatments.

Besides contributing to the advancement of knowledge for this plant species under specific light spectra, this experiment paves the way for further studies, not only in order to improve the cultivation of this plant and the production of metabolites, but also to deepen the study of biosynthetic pathways.

## 2 Materials and methods

### 2.1 Setting of environmental conditions and environmental monitoring system design

Due to the research aims, the trial was performed in a greenhouse in order to maintain proper environmental conditions as well as ensure their homogeneity among the treatments.

**Fig 1:**
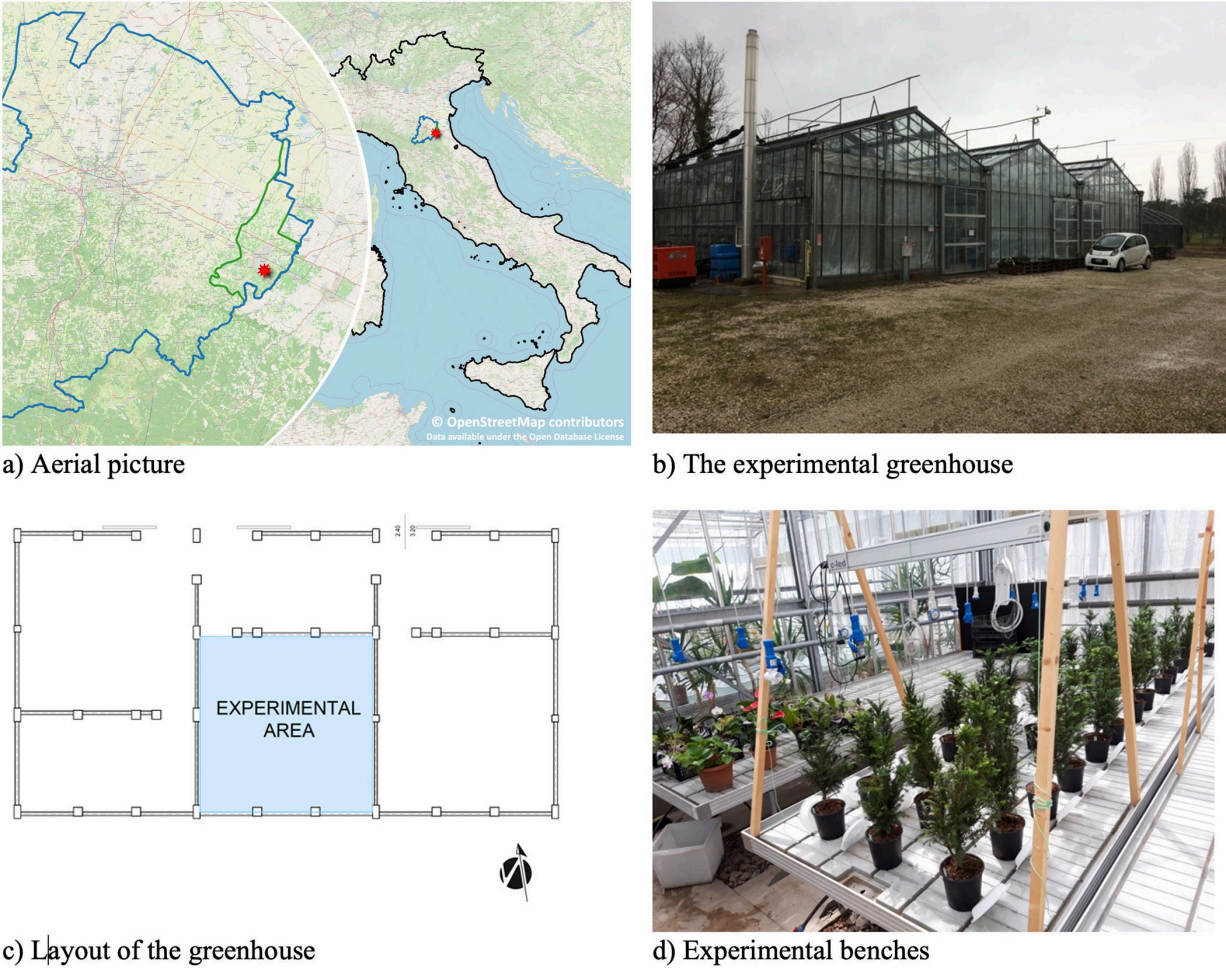
University of Bologna experimental greenhouse. a) shows the greenhouse location (red star). Black line represents the Italian border, blue line the Municipality of Bologna, the green line the Municipality of Imola. (© OpenStreetMap contributors. Data available under the Open Database License) b) The experimental greenhouse (outside view) c) The location of the experimental area in the greenhouse d) the experimental benches during the trial.

The trial took place in the experimental greenhouses of the Imola District of the University of Bologna (40 km South-East of Bologna) between the 19th of August 2020 and the 9th of December 2020. The greenhouse (see Fig 1) is divided into three spans and the trial was performed in the central one that covers an area of 8 m x 8 m; the height of the ceiling is between 4 and 5.5 m (measured respectively at eave and ridge). The facility is NE oriented (main axis azimuth 55°). Each span is provided with heating and cooling systems able to keep the temperature within the set range. Moreover, automatic openings help the temperature control and the air circulation. Finally, the greenhouse is provided with moveable shading screens useful to reduce the solar gain, to keep the indoor temperature and homogenize the indoor illumination. For the purposes of this experiment, according to Ellenberg [40], the heating and cooling systems were set to keep the temperature within 15°C and 25°C and the screens were kept throughout the trial.

To ensure the homogeneity of environmental conditions, specific tests were carried out before and during the trial. These tests allowed us to prove that the only difference in environmental conditions among the treatments was caused by the LED lamps in terms of intensity (PAR, Photosynthetically Active Radiation) and/or spectrum (red/blue ratio); tests were also intended to provide information on the environmental conditions during the experiment.

It is worth to recall that the LED lights were added as a supplement to the natural light, so the first test was designed to ensure that each treatment spot would receive the same amount of natural light. Considering that artificial light was provided during the daylight hours, the test on the natural light was performed before the trial started (August 2019), when the lamps were off. In a 5-day campaign, illuminance in each spot was measured every day, three times a day (at 9 am, 1 pm and 6 pm). Sensors recorded values between 500 and 6500 lux; these values are higher than the compensation point for *T*. *baccata* [40]. Differences between the values on the spots and the average were lower than 5% for each set of measures. Therefore, the natural light could be considered uniform for all spots. Under this light, the illuminance recorded by the sensor placed in the control spot could be assumed to be the natural light in the whole greenhouse span at the experiment level (70 cm above the floor level).

The artificial light was produced by five LED lamps with three emission spectra: blue (B), red (R) and mixed red and blue light (M) (see the “Light treatments” 2.3 section for more details). To produce the intensity required by the design of the trial, the lamp height was set in order to achieve the desired values 45 cm above the bench surface (average height of plants + pot at the beginning of the trial). Once the heights were set, the illuminance of each lamp was measured at foliage/canopy level for each treatment in total absence of natural light. Results are shown in Table 1.

**Table 1:** LED light characterization for each treatment.

	Treatment 1	Treatment 2	Treatment 3	Treatment 4	Treatment 5	Treatment 6
LED Lamp	M	M	M	R	B	0
PAR [PPFD]	150	100	50	100	100	0
Illumin [lx]	2270	1870	1390	1830	1170	0

Unlike illuminance, temperature and relative humidity are not affected by the artificial light (a preliminary test proved that the heat generated by the lamp was negligible), therefore we were able to monitor their values throughout the trial.

The analysis of recorded data allowed us to characterize the trial environmental conditions as well.

**Fig 2:**
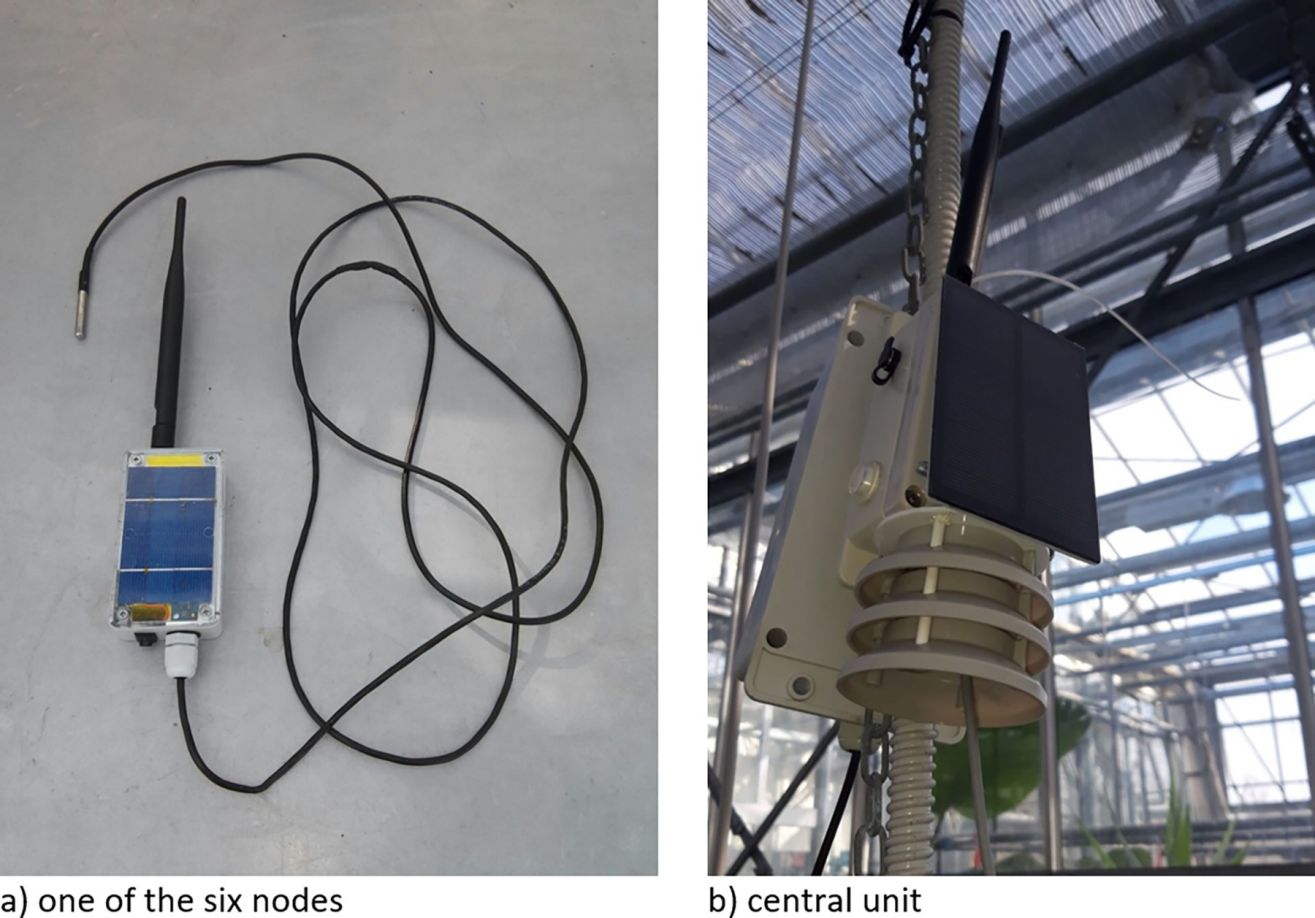
Node and central unit of the monitoring system.

To run the monitoring, besides the facility system, an additional monitoring equipment was placed in the greenhouse during the trial. This equipment–specifically designed and built for this experiment–was constituted by six nodes and a gateway (central unit) depicted in Fig 2.

Each node was placed in each treatment spot and measured temperature, relative humidity and illuminance every 3 minutes throughout the trial. The collected data were wirelessly sent to the gateway that uploaded data in a server with an internet connection allowing the experimental conditions to be checked remotely and in real time. Finally, data were stored and analyzed.

The sensor data characteristics and specification are reported in Table 2.

**Table 2:** Table of sensor characteristics. Resolution and accuracy of the sensors used for the monitoring activity.

Sensor	Resolution	Accuracy
Temperature	0.04°C	± 0.3°C
Relative Humidity	0.7%	± 2%
Illuminance	1 lx	± 1%

The homogeneity analysis was based on Barbaresi et al. [41], who designed a method to divide a room into zones with the same temperature and humidity. The proposed method was later implemented to assess the homogeneity of indoor conditions in a subsequent work [42].

The above-mentioned paper assesses the homogeneity of environmental conditions by comparing data collected in a monitoring campaign with two reference parameters, IRV and ARV. The IRV–Identity Reference Value–is calculated on the sensor characteristics and is used to define when temperatures and humidity values can be considered identical. The second parameter–the Acceptable Reference Value–is based on the purposes of the specific research and is used to define when differences in environmental conditions can be considered acceptable; in other words, when we can assume that the zones monitored by the sensors are in the same environmental conditions.

In accordance with the two above-mentioned studies, the IRVs are set as the sum of sensors’ resolution and accuracy. On the other side, the ARVs are defined according to Ellenberg [40] and to the heating and cooling systems performances. Then, for the purpose of this study, IRV and ARV were set as follows (see Table 3):

**Table 3:** IRV and ARV values. Values of temperature and relative humidity assumed as thresholds for the uniformity of greenhouse environmental conditions.

	IRV	ARV
Temperature	0.7°C	2°C
Relative Humidity	4.7%	5%

It is worth to note that the ARVs are remarkably lower than differences that can occur in *T*. *baccata* natural sites. According to the methods reported in [41], the following procedure was applied to temperature and humidity records:

- temperature and relative humidity in each treatment were monitored every 3 minutes throughout the trial;
- Data collected in a treatment were cleaned and compared to data collected in the other treatments, and differences were calculated for every record;
- The value *z*_*ij*_ *= |m*_*ij*_*|+sd*_*ij*_ was calculated for every pair of sensors, where *i* and *j* are two sensors out of the six, *m* and *sd* the respective mean and standard deviation of the differences;
- The *z* values were finally compared to IRV and ARV. In order to state if all the treatments were under the same environmental conditions, all the *z* values for both temperature and humidity should be lower than ARV.

### 2.2 Plant material

Forty European yew (*Taxus baccata* L.) plants were purchased from a large commercial nursery (Vivai Guagno, Santa Giustina in Colle, Padua, Italy) (45.55435 N, 11.92062 E, 25 m above sea level) to be used for the trial in the experimental greenhouse. In the nursery, the plants had been individually grown from seed up to five years of age in 3 L black plastic pots (height 15 cm, superior internal diameter 16 cm) with a mixed peat-coconut fiber-pumice substrate ensuring good homogeneity across pots. At the beginning of the experiment (19 August 2019), plants averaged 52.5 ± 6.3 cm height; their individual morphology was quite heterogeneous, making it necessary to assign them to the different groups as described in the following point.

### 2.3 Experimental design

Each plant was randomly labelled, and its weight, height, and number of sprouts were measured. Prior to weighing, the plants were well watered and let drain in order to minimize differences due to disparity in substrate humidity. Taking into account the differences for the three measured parameters, 36 plants were distributed into 6 groups so that the internal heterogeneity of each group was similar, making the groups comparable. The resulting group composition was as follows:

Group 1: plants n. 1, 2, 18, 19, 28, 33
Group 2: plants n. 3, 21, 22, 23, 31, 32
Group 3: plants n. 16, 24, 25, 26, 34, 36
Group 4: plants n. 11, 12, 20, 27, 29, 30
Group 5: plants n. 5, 7, 8, 9, 13, 14
Group 6: plants n. 4, 6, 10, 15, 17, 35

The four remaining plants were sampled for chemical analysis at time zero (T0; 19 August 2019), shortly before the start of LED lighting.

### 2.4 Light treatments

Each group of six plants was exposed to a top-light LED lamp emitting a different combination of light spectrum and intensity (PPFD, Photosynthetic Photon Flux Density), as follows:

Group 1: R/B/Fr LED (2/3 red, 1/3 blue), PPFD 150 μM m^-2^ s^-1^ (R/B/Fr 150)
Group 2: R/B/Fr LED (2/3 red, 1/3 blue) PPFD 100 μM m^-2^ s^-1^ (R/B/Fr 100)
Group 3: R/B/Fr LED (2/3 red, 1/3 blue) PPFD 50 μM m^-2^ s^-1^ (R/B/Fr 50)
Group 4: Red LED, PPFD 100 μM m^-2^ s^-1^ (R 100)
Group 5: Blue LED, PPFD 100 μM m^-2^ s^-1^ (B 100)
Group 6: natural light control (Ctrl)

(R = Red, B = Blue, Fr = Far Red)

### 2.5 Plant cultivation

Plants were grown under treatment from 19 August to 9 December 2019. The supplemental lighting had the same light period of 14 hours per day for all LED treatments. The natural light varied according to the season (see Table 6), while the indoor temperature, and consequently relative humidity, varied always within the set range. Watering was done when needed; since European yew is a slow-growing plant, no fertilization was given during the trial, in order to exclude morpho-physiological responses due to factors other than LED light treatment. Moreover, in order to give each plant canopy a uniform light exposure, all plants were rotated a quarter turn every three days.

### 2.6 Chemicals

Deuterium oxide (D_2_O, 99.90% D), CD_3_OD (99.80% D) were purchased from Eurisotop (Cambridge Isotope Laboratories, Inc, France). Standard 3-(trimethylsilyl)-propionic-2,2,3,3-d_4_ acid sodium salt (TMSP), baccatin III ≥95% (HPLC), sodium phosphate dibasic anhydrous and sodium phosphate monobasic anhydrous and all the other chemicals and solvents were purchased from Sigma-Aldrich Co. (St. Louis, MO, USA).

### 2.7 Biometric surveys and sampling for metabolomic analysis

Morphometric/biometric surveys were performed once every 28 days at 0, 28, 56, 84 and 112 days after treatment (DAT), spanning from 19 August to 9 December 2019. At each survey three pictures per plant were taken for canopy shape evaluation, and the following parameters were measured:

- plant height: measured from the crown to the tip of the main stem; branches occasionally taller than the main stem were not considered;
- sprout number: number of young sprouts having pale green color;
- NDVI (Normalized Difference Vegetation Index), which is defined as the ratio between the difference and the sum of reflectance values in the near-infrared and red spectrum, was selected as indicator of plant growth status. It was determined with the portable GreenSeeker reader (Trimble Inc., Sunnyvale, CA; USA), holding the instrument horizontally 50 cm above plant tips.

### 2.8 Metabolomic analysis

For metabolomic analysis, samples from each plant were collected every 28 days by cutting 3 representative branches per individual. The samples were dried at 40°C and powdered using an electrical grinder (IKA, A11 basic, Merck, Italy).

Plants extracts were prepared according to Mandrone et al. [43] with slight modifications. Briefly, thirty mg of each sample were extracted with 1 mL of mixture (1:1) of phosphate buffer (90 mM; pH 6.0) in H_2_O-*d*2 (containing 0.01% TMSP) and MeOH-*d*4 by ultrasonication (TransSonic TP 690, Elma, Germany) for 20 minutes. After this procedure, samples were centrifuged for 10 min (17000 x *g*), then 700 μL of supernatant were transferred into NMR tubes.

### 2.9 NMR measurement

^1^H NMR spectra were recorded at 25°C on a Varian Inova 600 MHz NMR instrument (600 MHz operating at the ^1^H frequency) equipped with an indirect triple resonance probe. Methanol-*d*_*4*_ was used for internal lock. Each ^1^H-NMR spectrum consisted of 256 scans (corresponding to 16 min) with the relaxation delay (RD) of 2 s, acquisition time 0.707 s, and spectral width of 9595.8 Hz (corresponding to δ 16.0). A presaturation sequence (PRESAT) was used to suppress the residual water signal at δ 4.83 (power = -6dB, presaturation delay 2 s). The spectra were manually phased, and baseline corrected and calibrated to the internal standard trimethyl silyl propionic acid sodium salt (TMSP) at δ 0.0 using Mestrenova software (Mestrelab Research, Spain). For metabolomic analysis, the regions of δ 5–4.5 and δ 3.34–3.30 were excluded because of the residual solvent signals. Then spectral intensities were reduced to integrated regions of equal width (δ 0.04) corresponding to the region from d 0.0 to 10.0 and normalized by total area.

The spectrum of baccatin III was recorded in methanol-*d*_*4*_.

### 2.10 Data management and multivariate data analysis

Biometric data were split between two treatment sub-groups: one at different light intensity, composed by Ctrl, R/B/Fr 50, R/B/Fr 100 and R/B/Fr 150; and another at different light quality, composed by R 100, B 100 and R/B/Fr 100. In the two sub-groups, data were submitted to one-way ANOVA at each of the five surveys, using the STATISTICA v.10.0 software (StatSoft, Tulsa, OK, USA). For selected traits, the time trends of the treatments and their 95% confidence bands were traced, using the SigmaPlot 10 software (Systat Software Inc., San José, CA, USA).

The SIMCA software (v. 16.0, Umetrics, Sweden) was used for multivariate data analysis. In particular, data were subjected to Pareto scaling and supervised models were evaluated by the goodness of fit (R^2^x (cum) and R^2^y(cum)) and goodness of prediction (Q^2^(cum)), together with the parameters given by cross validation tests: permutation test (performed using 200 permutations) and CV-ANOVA.

## 3 Results

For temperature and humidity characterization, the method described in Section 2.1 was applied and data measured by the sensors for each experimental treatment were compared. Tables 4 and 5 show the results.

**Table 4:** Analysis of the temperatures measured in each treatment. Data are expressed in Celsius degree.

Treatment	Mean	St.dev.	|m|+sd
T1 vs T2	0.314	0.960	1.274
T1 vs T3	0.242	1.030	1.272
T1 vs T4	0.271	0.296	0.567
T1 vs T5	0.285	0.618	0.903
T1 vs T6	0.650	0.518	1.169
T2 vs T3	-0.071	0.301	0.372
T2 vs T4	-0.009	0.980	0.989
T2 vs T5	-0.025	0.464	0.489
T2 vs T6	0.320	0.785	1.105
T3 vs T4	0.067	1.095	1.161
T3 vs T5	0.049	0.569	0.618
T3 vs T6	0.437	0.834	1.271
T4 vs T5	-0.004	0.648	0.651
T4 vs T6	0.324	0.528	0.852
T5 vs T6	0.353	0.530	0.882

**Table 5:** Analysis of the relative humidity measured in each treatment. Data are expressed in percentage.

Treatment	Mean	St.dev.	|m|+sd
T1 vs T2	-0.196	2.845	3.041
T1 vs T3	-0.911	3.017	3.927
T1 vs T4	-1.106	1.687	2.793
T1 vs T5	-0.753	2.076	2.830
T1 vs T6	-1.440	2.426	3.867
T2 vs T3	-0.768	1.442	2.210
T2 vs T4	-1.037	2.612	3.650
T2 vs T5	-0.589	1.677	2.267
T2 vs T6	-1.137	2.315	3.452
T3 vs T4	-0.276	3.004	3.280
T3 vs T5	0.169	1.853	2.021
T3 vs T6	-0.435	2.566	3.001
T4 vs T5	0.348	2.183	2.531
T4 vs T6	-0.229	2.421	2.650
T5 vs T6	-0.592	1.942	2.534

The results show that the mean of differences was very low, even under the IRV in some cases and in general confirm the homogeneity of temperature and humidity. Under this light, to characterize the environmental conditions of the trial, the average temperature and humidity of the six treatments were retained to define the environmental conditions for all the treatments.

As reference, Table 6 reports the significant environmental conditions that characterized four periods during the trial. Each period is defined as the time between a morphometric/biometric survey and the following one: Period 1 (19/08–16/09), Period 2 (17/09–14/10), Period 3 (15/10–11/11) and Period 4 (12/11–09/12).

**Table 6:** Indoor environmental data. Characterization of temperature, relative humidity and natural light (average values) measured in the four periods of the experiment.

	Period 1		Period 2		Period 3		Period 4	
	day	night	day	night	day	night	day	night
T [°C]	23.65	21.44	22.87	20.05	20.55	16.61	19.42	15.04
rH [%]	58.07	70.03	63.62	70.78	70.00	77.77	56.47	64.71
Nat. light [lx]	1495	-	518	-	299	-	1280	-

### 3.1 Growth analysis

**Fig 3:**
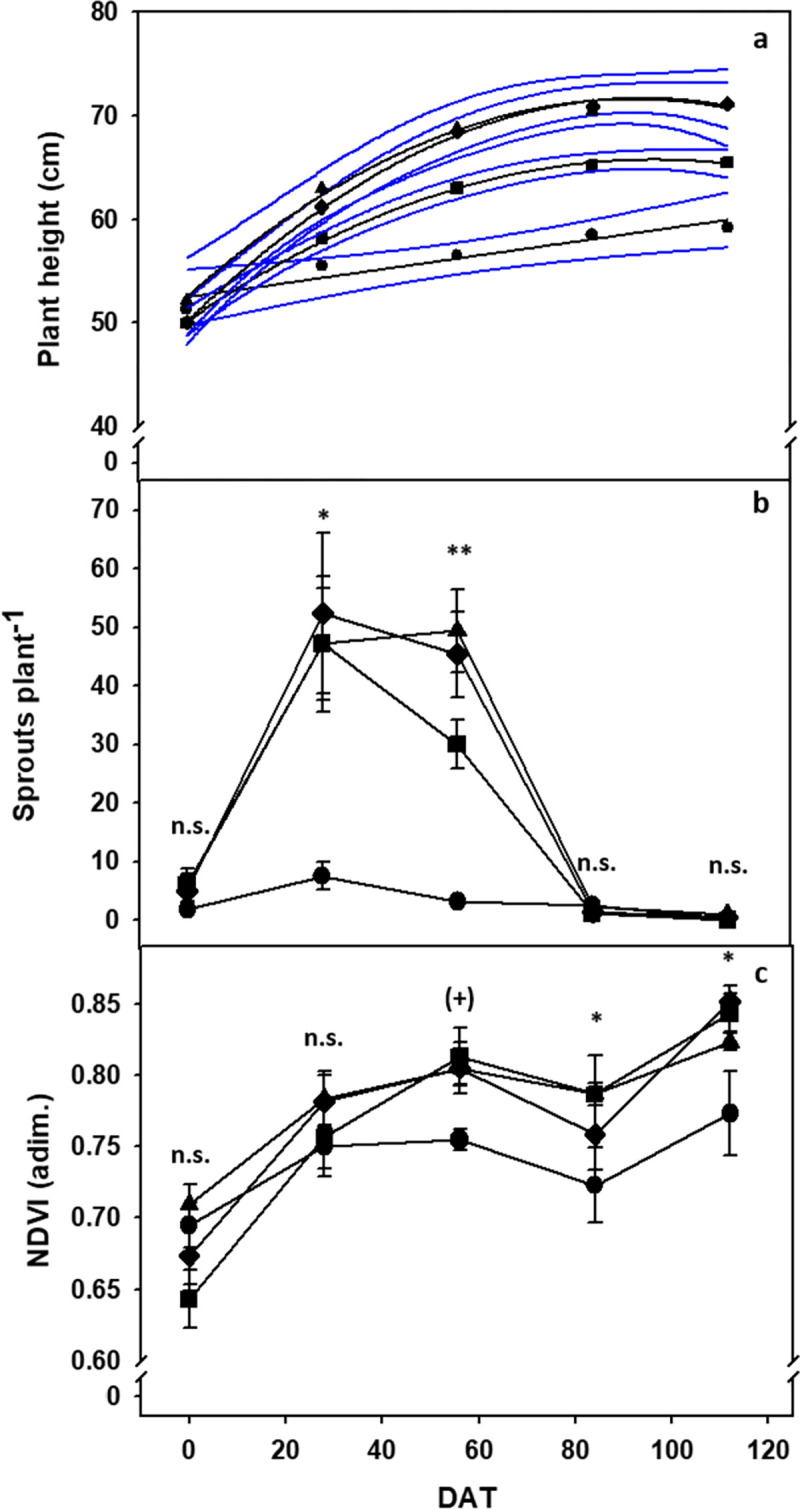
Morphology analysis. Plant height (a), number of sprouts per plant (b), and normalized difference vegetation index (NDVI) (c) at various days after treatment (DAT) in Taxus baccata exposed to different light intensity. Circles, squares, diamonds and triangles indicate the control and the R/B/Fr LED lamps at 50, 100 and 150 μM m^-2^ s^-1^, respectively. In plant height, the 95% confidence bands for the time trends of the four treatments are shown; in the other two traits, vertical bars indicate ± SE (n = 6); n.s., ^(+)^, * and ** indicate non-significant and significant at P ≤ 0.10, P ≤ 0.05 and P ≤ 0.01, respectively.

In the light intensity sub-group, plant height described a different trend across the 112 DAT (Fig 3A). Plants under natural light (Ctrl group) only modestly increased in height, whereas plants at increasing light intensity (R/B/Fr 50, 100 and 150 μM m^-2^ s^-1^) increased to a larger extent: the two upper levels (100 and 150 μM m^-2^ s^-1^) were practically superimposed, while the lower level (50 μM m^-2^ s^-1^) traced an intermediate trend between Ctrl and the two upper levels.

The number of sprouts per plant remained very low under natural light, whereas it was strongly enhanced by the LED treatments in the early phase (28 and 56 DAT) (Fig 3B). The lower light intensity (50 μM m^-2^ s^-1^) showed slightly lower data than the two upper light intensities (100 and 150 μM m^-2^ s^-1^) only at 56 DAT. In the later growth phase (84 and 112 DAT), the stimulus to sprouting ceased, and all treatments fell very close to zero sprouting.

The NDVI exhibited non-significant differences among light intensity levels until 28 DAT (Fig 3C). Thereafter, treatment differences became statistically significant, indicating that the Ctrl treatment was always below the three LED treatments. No further difference was shown among these latter.

In the light quality sub-group, no significant difference in the three biometric traits was evidenced, apart from the number of sprouts at 84 DAT. In general, a slight beneficial influence is perceived for the blue light, alone or in combination with the red and far red (B 100 and R/B/Fr 100), over the red light (R 100) in sprouts per plant (Table 7), whereas in plant height (Table 8) and NDVI (Table 9) this effect is unperceivable.

**Table 7:** Number of sprouts per plant at various days after treatment (DAT) in Taxus baccata exposed to different light quality (R, red; B, blue; Fr, far red) at 100 μM m-2 s-1. Data are means ± SE (n = 6).

DAT	0	28	56	84	112
R 100	1.5 ± 1.0	25.2 ± 6.4	28.0 ± 4.4	3.7 ± 0.3 a	1.0 ± 0.4
B 100	2.0 ± 0.7	57.3 ± 10.5	34.8 ± 4.3	1.2 ± 0.7 b	0.2 ± 0.2
R/B/Fr 100	5.0 ± 0.9	52.3 ± 13.7	45.3 ± 7.4	1.3 ± 0.5 b	0.5 ± 0.5
Stat. signif.	n.s.	n.s.	n.s.	**	n.s.

**Table 8:** Plant height (cm) at various days after treatment (DAT) in Taxus baccata exposed to different light quality (R, red; B, blue; Fr, far red) at 100 μM m^-2^ s^-1^. Data are means ± SE (n = 6).

DAT	0	28	56	84	112
R 100	55.7 ± 0.9	60.3 ±1.4	65.0 ± 2.0	66.2 ± 1.2	66.7 ± 1.0
B 100	56.7 ± 2.0	61.7 ± 1.9	67.0 ± 1.9	69.7 ± 2.0	70.3 ± 2.3
R/B/Fr 100	50.0 ± 2.3	61.2 ± 4.6	68.5 ± 5.1	70.8 ± 5.4	71.2 ± 5.2
Stat. signif.	n.s.	n.s.	n.s.	n.s.	n.s.

**Table 9:** Normalized difference vegetation index (adim.) at various days after treatment (DAT) in Taxus baccata exposed to different light quality (R, red; B, blue; Fr, far red) at 100 μM m-2 s-1. Data are means ± SE (n = 6).

DAT	0	28	56	84	112
R 100	0.67 ± 0.02	0.78 ± 0.03	0.81 ± 0.01	0.75 ± 0.01	0.83 ± 0.01
B 100	0.68 ± 0.02	0.77 ± 0.02	0.84 ± 0.01	0.74 ± 0.03	0.83 ± 0.02
R/B/Fr 100	0.67 ± 0.02	0.78 ± 0.02	0.81 ± 0.02	0.76 ± 0.02	0.85 ± 0.01
Stat. signif.	n.s.	n.s.	n.s.	n.s.	n.s.

**Fig 4:**
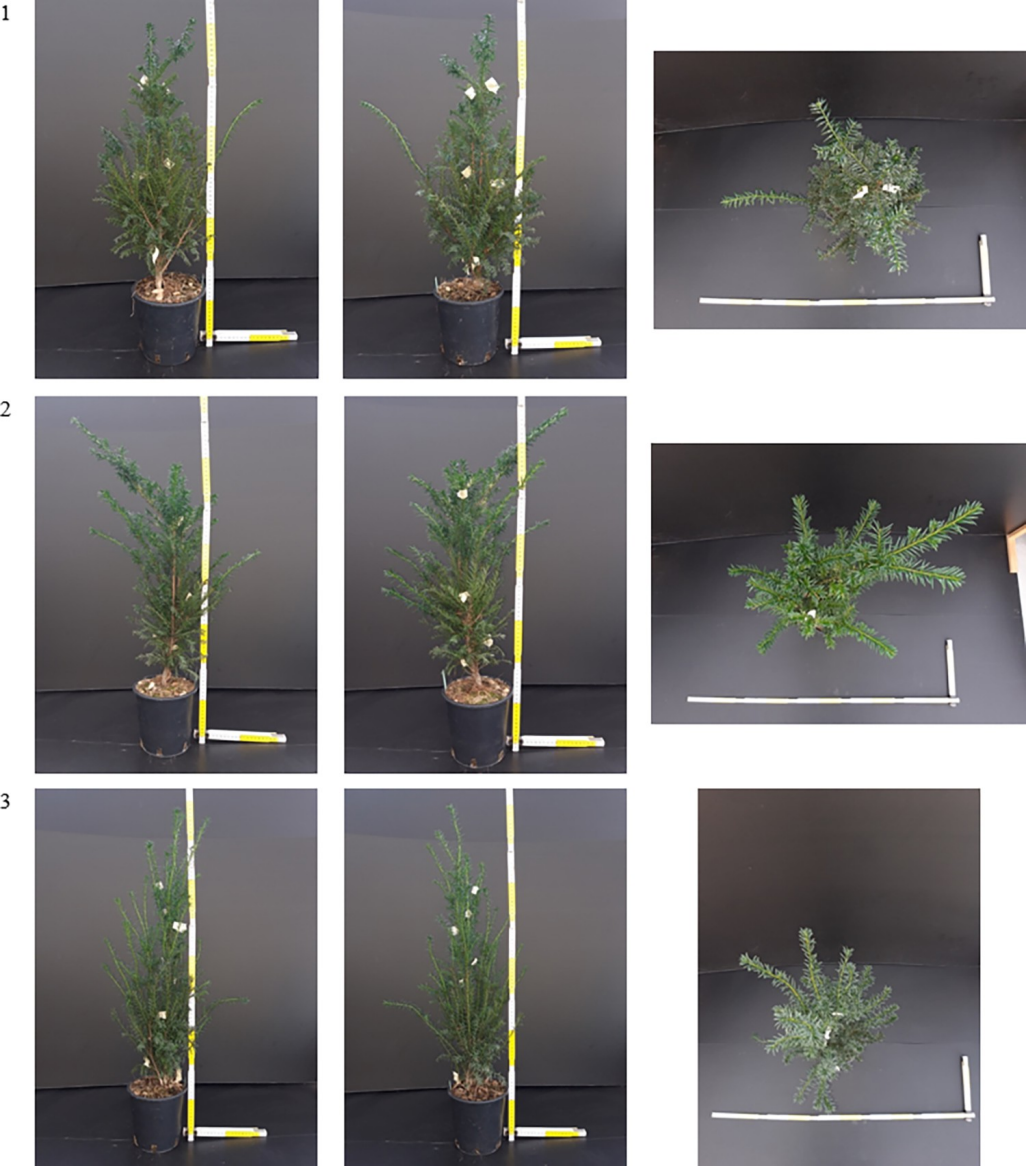
Plants’ views. Front, rear and above views of a plant from Control (1), R/B/Fr 100 (2), and B 100 (3) treatments on 9 December 2019.

The pictures in Fig 4 allow morphological differences to be detected among the different light treatments. No significant evidence of systematic differences was found. Individual variability within each group was as high as variability between different groups.

### 3.2 Metabolomic analysis

**Fig 5:**
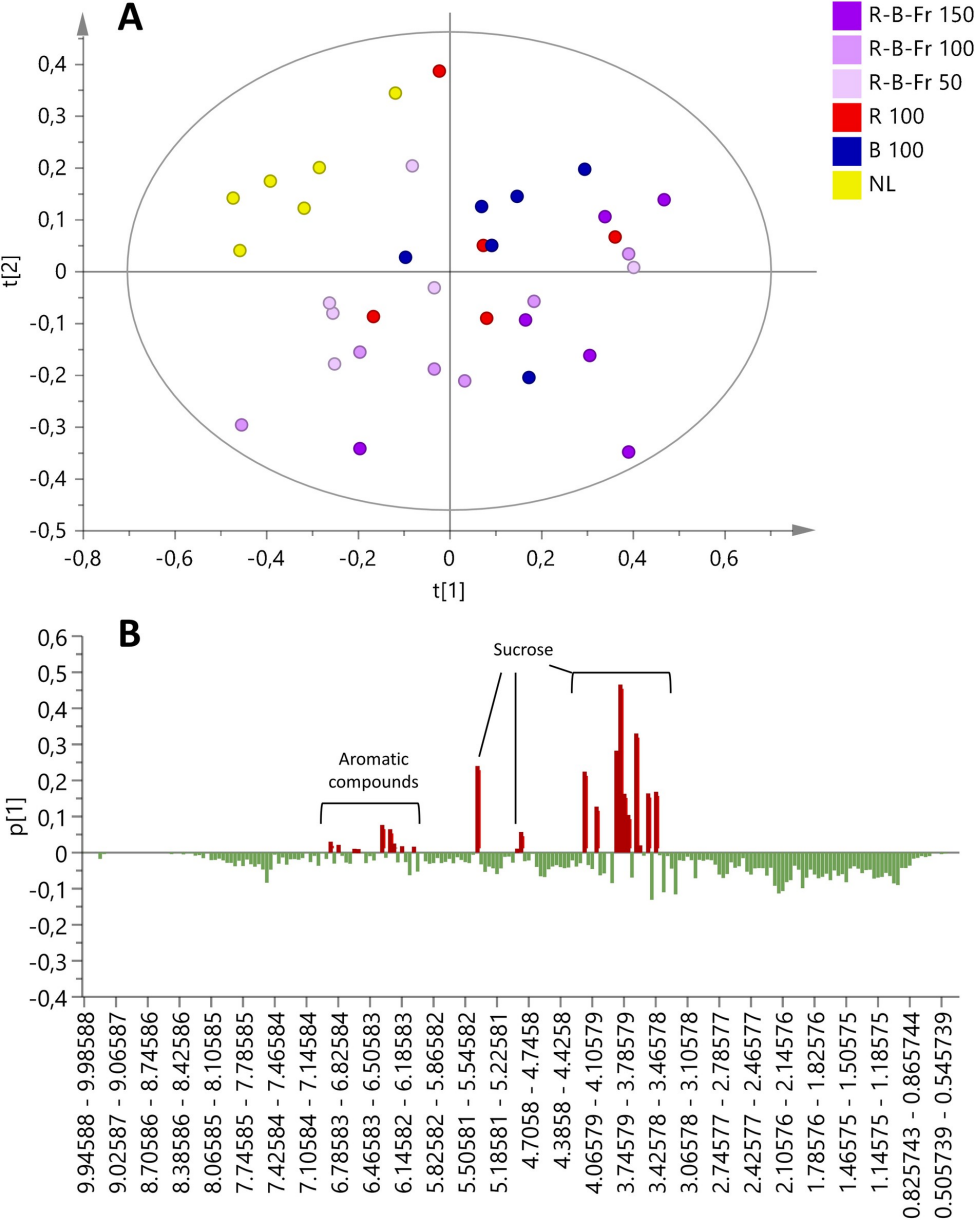
^1^H NMR based principal component analysis of T. baccata samples under different LED light treatments. A) Score scatter plot B) Loading column plot. R^2^(cum) = 0.552, Q^2^(cum) = 0.442.

In order to detect eventual variations in the metabolome of the samples at 28 DAT, Principal Component Analysis (PCA) was performed on bucketed ^1^H NMR spectra, used as *x* variables of the model. As shown by the score scatter plot (Fig 5A), samples of the control group (NL) were clustered in the upper left quadrant (negative component t [1] and positive component t [2]).

As shown by the loading plot (Fig 5B), some spectral signals, resonating in the aromatics and carbohydrates regions, were particularly intense in the treated samples, determining a shift along the positive component t [1] of these latter in the PCA score scatter plot (Fig 5A). In fact, it was evident from the ^1^H NMR spectra that treated samples were characterized by an increase in sucrose concentration compared to the control group. In addition, some spectral signals resonating at δ 6.26, 6.34, 6.78, 6.86 (aromatic region) were found characteristic of the treated samples. Even though the plants belonging to the same group showed individual responses to the treatment, group 1 (treatment R/B/Fr 150 μM m^-2^ s^-1^) resulted to be the most different from the control group (natural light, NL), while group 3 (treatment R/B/Fr 50 M m^-2^ s^-1^) resulted more similar to the control.

**Fig 6:**
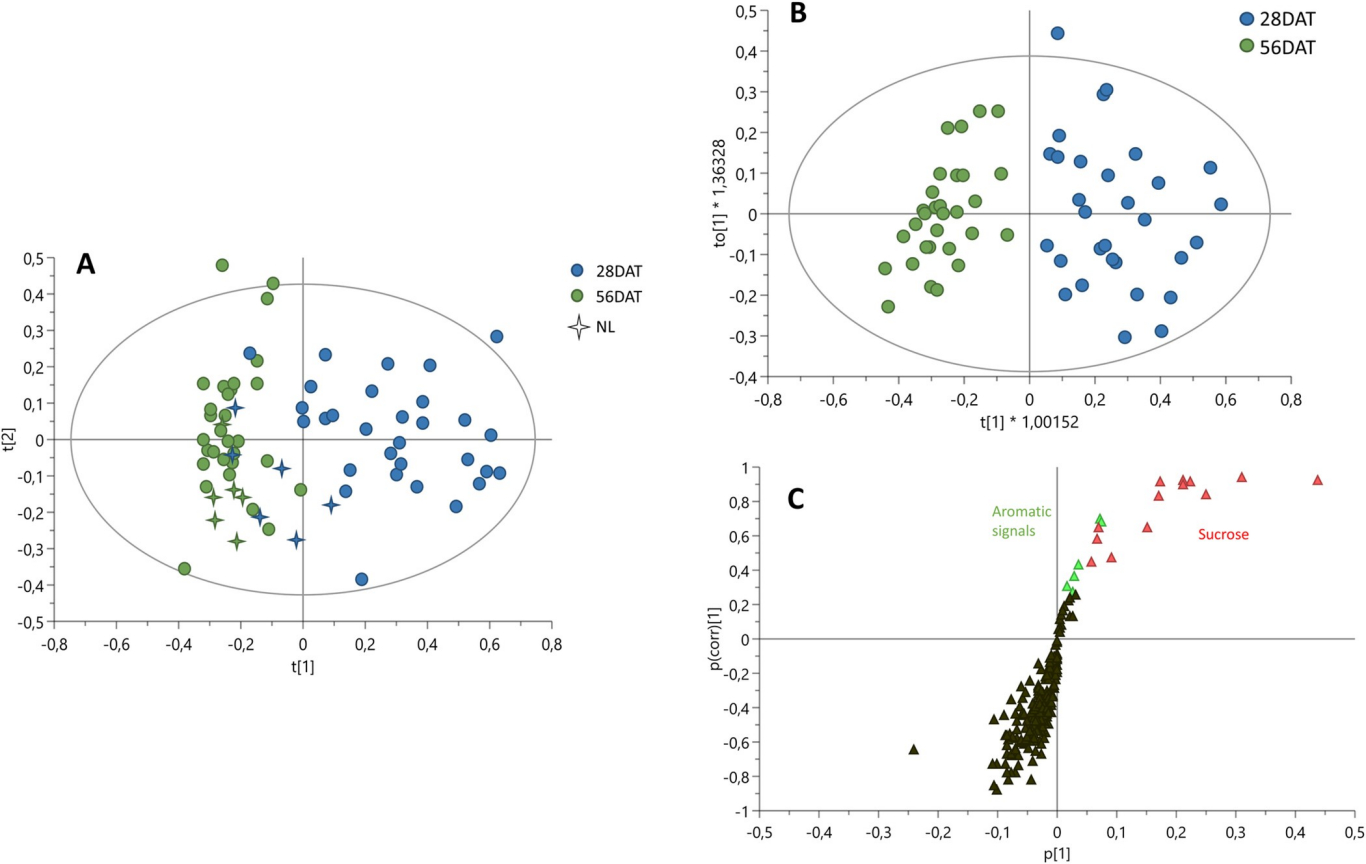
A) ^1^H NMR based PCA of T. baccata samples under different LED light treatments at 28 days and 56 days since treatments started. R^2^(cum) = 0.871, Q^2^(cum) = 0.674. B) Score scatter plot of OPLS-DA model using sampling time as discriminant classes. C) S-plot of OPLS-DA model.

According to the results of the metabolomic analysis on samples at 56 DAT, in this stage the treated plants were no longer different from the control group. The PCA model built on the bucketed ^1^H NMR spectra of both 28 DAT and 56 DAT (Fig 6A), showed that all samples at 56 DAT were close to the control group at 28 DAT. Only the treated samples at 28 DAT showed evident differences compared to the controls.

In order to obtain more information on the metabolites responsible for the separation of the treated samples at the different time points, OPLS-DA (Orthogonal Partial Least Squares-Discriminant Analysis) was carried out on bucketed ^1^H NMR spectra of treated plants, using as discriminant classes the two time points of sampling (control plants were excluded from the analysis) (Fig 6B and 6C). The model found a perfect fit to the response using two components, with goodness of fit (*R*^*2*^*y*(cum)) of 82% and goodness of prediction (*Q*^*2*^(cum)) of 71%. The model was evaluated by permutation test (performed using 200 permutations), which gave *R*^*2*^(cum) of 83% and intercept on *y* axis of *R*-line was 0.16, and *Q*^*2*^(cum) of 71% and intercept on *y* axis of *Q*-line was -0.29. Moreover, the significance of the model was tested by the ANOVA of cross-validated residuals (CV-ANOVA) giving *p* = 2.1 x 10^−14^ and *F* = 34. The results showed by the *S*-plot of this model (Fig 6C) were consistent with the results of the PCA, confirming that at 28 DAT the treatments determined an increase in sucrose and aromatic compounds. In addition, from this plot it resulted that all the other metabolites were more abundant at 56 DAT.

**Fig 7:**
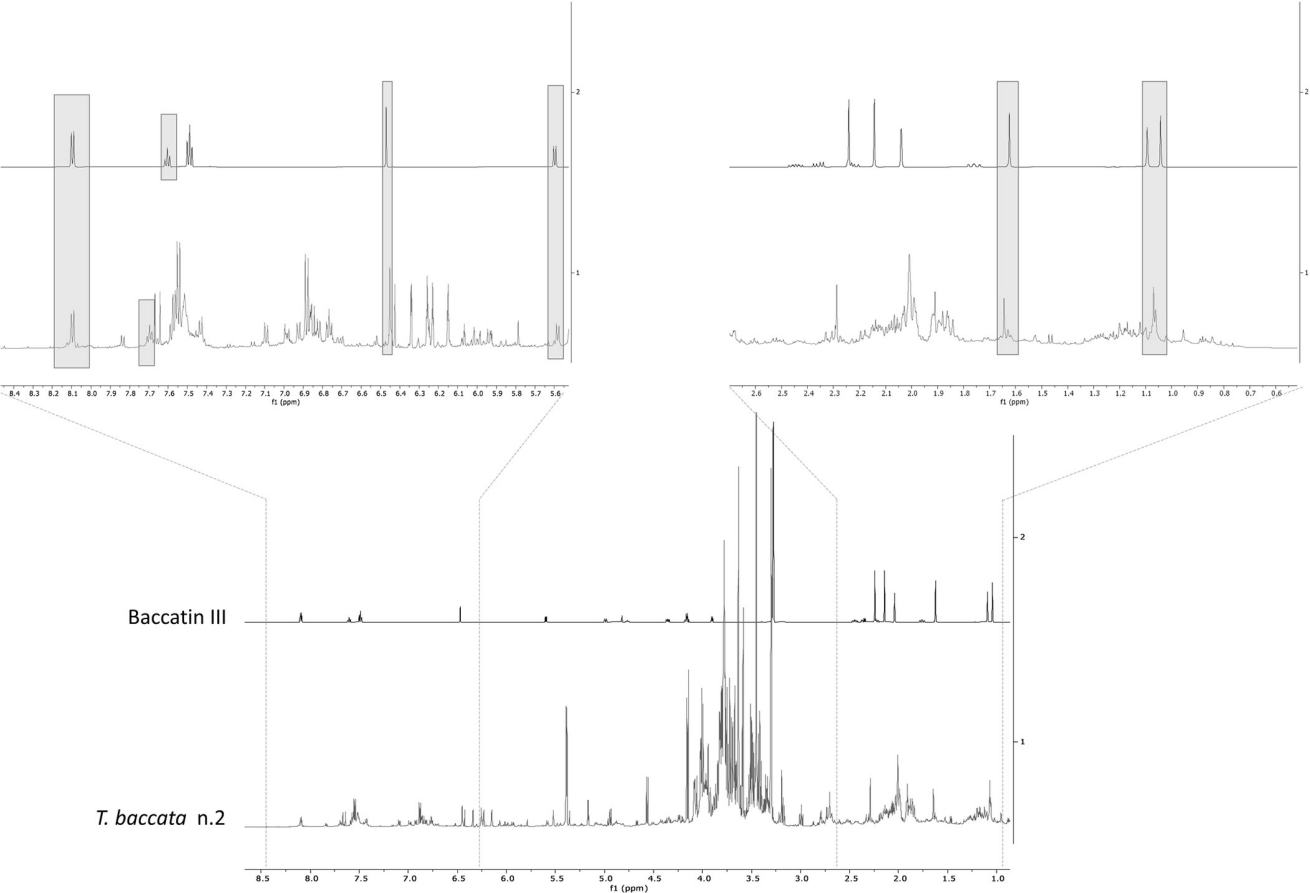
Comparison between ^1^H NMR spectra of baccatin III and plant number 2 at 56 DAT. On top are given extended regions from δ 0.6 to 2.6 and from δ 5.5 to 8.4.

Spectral signals ascribable to taxanes were also found in the ^1^H NMR profiles of the experimental plants, which were compared to the ^1^H NMR spectrum of baccatin III, a precursor of taxol [44] (S1 Fig, Fig 7). These signals were scarcely affected by the treatment. However, from the S-plot of the OPLS-DA, it was possible to detect a slight increase of the putative taxane in plants at 56 DAT (S2 Fig).

## 4 Discussion

To our best knowledge, the above results on *Taxus baccata* morphology, growth and metabolomic profile in response to differential LED treatments are the first to show up in the literature. In fact, coniferous plants are seldom surveyed among medicinal and aromatic plants, as a recent review demonstrates [17]. Still to our best knowledge, the only two *Taxus* species having been focused in a previous LED experiment were *Taxus yuannensis* [27] and *Taxus chinensis* [45], and in the latter case only plant growth and net photosynthetic rate were assessed in response to LED supplementation during the winter time.

In this paucity of information, despite a relatively short experiment and the intrinsically slow growth of this species, supplemental LED illumination of moderate intensity (up to 150 μM m^-2^ s^-1^) was able to determine increased plant height, enhanced sprouting in the early growth phase, and somewhat better growth status (Fig 3). The temporal phase and the extent to which LED determined the above effects varied depending on light intensity (0–150 μM m^-2^ s^-1^ of supplemental PAR) as well as composition (R, B and R/B/Fr). This indicates that additional work is needed to better circumstantiate suitable plant conditions leading to consistent benefits from LED use.

However, the generally good effects obtained with LED lighting in both quantitative (plant height and number of sprouts) and qualitative (NDVI as indicator of vigor) growth, are the premise for sustained growth over the medium to long term, potentially leading to higher production of valuable metabolites. These effects increased when light intensity rose, although beyond 100 μM m^-2^ s^-1^ these effects faded, indicating that effective growth stimulus can be triggered by a small LED supplementation.

At the focal light intensity of 100 μM m^-2^ s^-1^, LEDs including the blue wavelength in their spectra (B and B/R/Fr) were shown to be slightly more effective than the monochromatic red light (R). This circumstance is not consistently echoed in the literature on LED use, although it should be born in mind that no specific work has so far addressed *Taxus baccata*.

Both the combination of natural and LED light, and the low intensity of supplementary lighting require to be carefully managed in order to exclude any confounding factor. Therefore, besides an accurate design of the light supplementation and environmental control systems, this study paid special attention to assure and prove the uniformity of environmental conditions throughout the experiment. A system capable of monitoring temperature, relative humidity, and illuminance, allowed us to map and characterize every experimental slot with a high spatial and temporal resolution. The analysis of collected data allowed us to confirm that the different plant responses had to be attributed only to LED light supplementation.

The metabolomic analysis showed that one of the most prominent effects exerted by LEDs on *T*. *baccata* was to determine an increase in the sucrose content. This is explained by the stimulation of the photosynthetic activity due to the additional lighting. Moreover, treated plants showed higher content of aromatic compounds, indicating that light exposure might have triggered the synthesis of photoprotective compounds such as phenols and flavonoids, which are generally found in the *Taxus* genus [46, 47].

The treatment at 50 μM m^-2^ s^-1^ was not sufficient to induce a strong variation in the most abundant metabolites produced by *T*. *baccata*. On the contrary, according to the PCA, plants treated with the highest intensity LEDs were the most diversified from the control group, indicating that light intensity has a strong impact on plant metabolome. Moreover, varying the light composition (red, blue and red/blue/far red) at intermediate intensity (100 μM m^-2^ s^-1^) did not determine significant differences in plant growth and morphology (Table 8). However, it may not be excluded that by varying some conditions, e.g., increasing light intensity, differences in light spectral composition might result in beneficial differences in plant behaviour.

The OPLS-DA model built using the ^1^H NMR data collected at two different time points highlighted that treated plants become more similar to the control group at 56 DAT, and by S-plot it was possible to observe a general increase of metabolite content during plant growth. Although the treatment was not affecting the putative taxane concentration, it was increasing the number of sprouts and height of the plants. According to this data, the treatment of taxus with LED lights, might lead to obtain a higher amount of plant material with a consequent increase in the recovery of the metabolites of interest.

## 5. Conclusions

Studying the effects of supplemental LED lighting on the growth of medicinal plants and the production of secondary metabolites of pharmacological interest is a topical and challenging subject, which is gaining interest by many scholars across the globe. Nevertheless, it still needs further investigations, especially as it concerns conifers, where the effects of LED treatments have mostly been studied in forest tree species, focusing on growth and physiology, and have largely been performed on tissue cultures when the target feature was the production of metabolites of pharmaceutical interest.

Therefore, this study addressed the challenge of investigating the effects of both quality and intensity of supplemental LED lighting on both growth and metabolism of *Taxus baccata*, known for being a source of substances with antitumor activity.

The research focused on a cultivation environment which exploits natural light, allowing lower LED lighting intensities to be used, compared to those that would be needed in indoor farming. This was made possible thanks to a continuous monitoring and control of environmental parameters, which allowed us to assure homogeneous conditions of base natural light, temperature, and humidity, and thus to refer the differences in plant growth and metabolism only to the different light treatments.

Supplemental LED treatments showed clear effects on plant growth, in particular in terms of increased plant height, number of sprouts, and plant vigor. Both unsupervised (PCA) and supervised (OPLS-DA) analyses were performed on ^1^H NMR profiles of samples, showing that sucrose and aromatic compounds were significantly higher in treated plants. The increment in sucrose production is likely reflecting the increased photosynthetic activity of plants exposed to LED lighting [48]. In addition, the increase of aromatic compounds might represent a photoprotective strategy [49]. An overall increase of the most abundant metabolites, including the detected putative taxane, was observed during plant growth. These results highlight the importance of paying attention to plant growth stage and treatment duration when setting greenhouse cultivation. The study allowed us to identify 100 μM m^-2^ s^-1^ as the light intensity threshold above which no significant benefits can be obtained, and to detect that LED spectra including both red and blue show better results. However, further research is needed to better investigate the effects of different light spectra, both obtained with different proportions of the wavelengths already considered or different wavelengths within and outside the visible (including UV, for example), and the effects of different intensities, also to evaluate how the extent of the effects in the short and longer term depends in a combined way on the spectrum, intensity and duration of the treatment. Moreover, since the advantages shown by the growth and metabolic parameters deriving from the light treatments have proved not to increase significantly or—in some cases—to decrease after a certain period, further research will focus on the effects of LED treatments at different durations and in different phases of plant growth. Finally, future developments of this research will be aimed at tracking the effects after the interruption of the light treatment, to assess effect persistence over time, in relation to the phase in which they are administered.

## Supporting information

S1 Fig ^1^H NMR spectrum of baccatin III. (TIF)

S2 Fig S-plot of OPLS-DA model where the bucket containing a signal ascribable to taxane (δ 8.10–8.14) was highlighted showing a slight increase in plants at 56 DAT. (TIF)

S1 Dataset (ZIP)
