# Supplementary figures and images for: Effects of LED supplemental lighting on the growth and metabolomic profile of Taxus baccata cultivated in a smart greenhouse
Source: PLoS One. 2022 Jul 8;17(7):e0266777. doi: 10.1371/journal.pone.0266777 (PMC9269924; doi:10.1371/journal.pone.0266777)

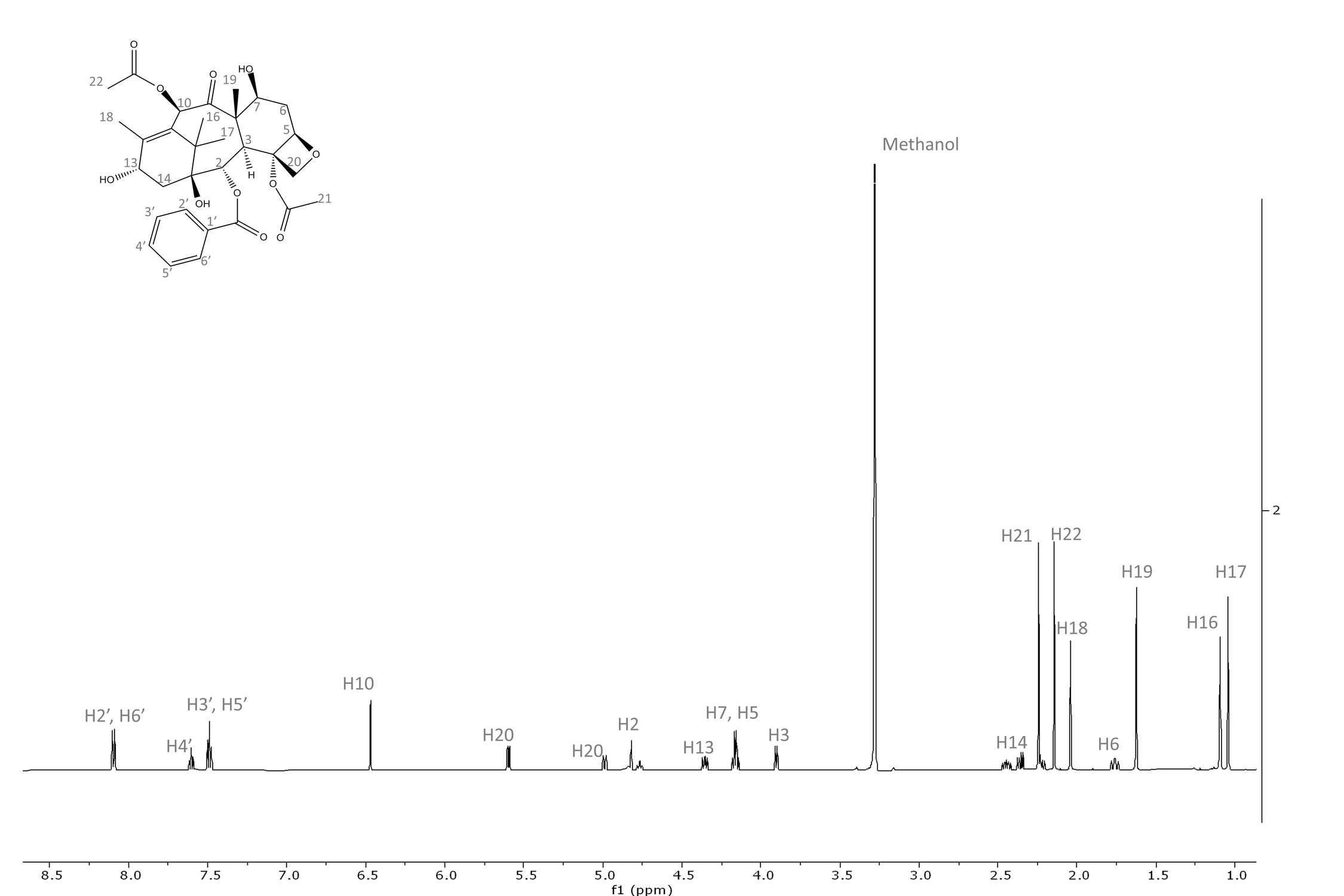

Supplement: S1 Fig — (TIF) [file pone.0266777.s001.tif]

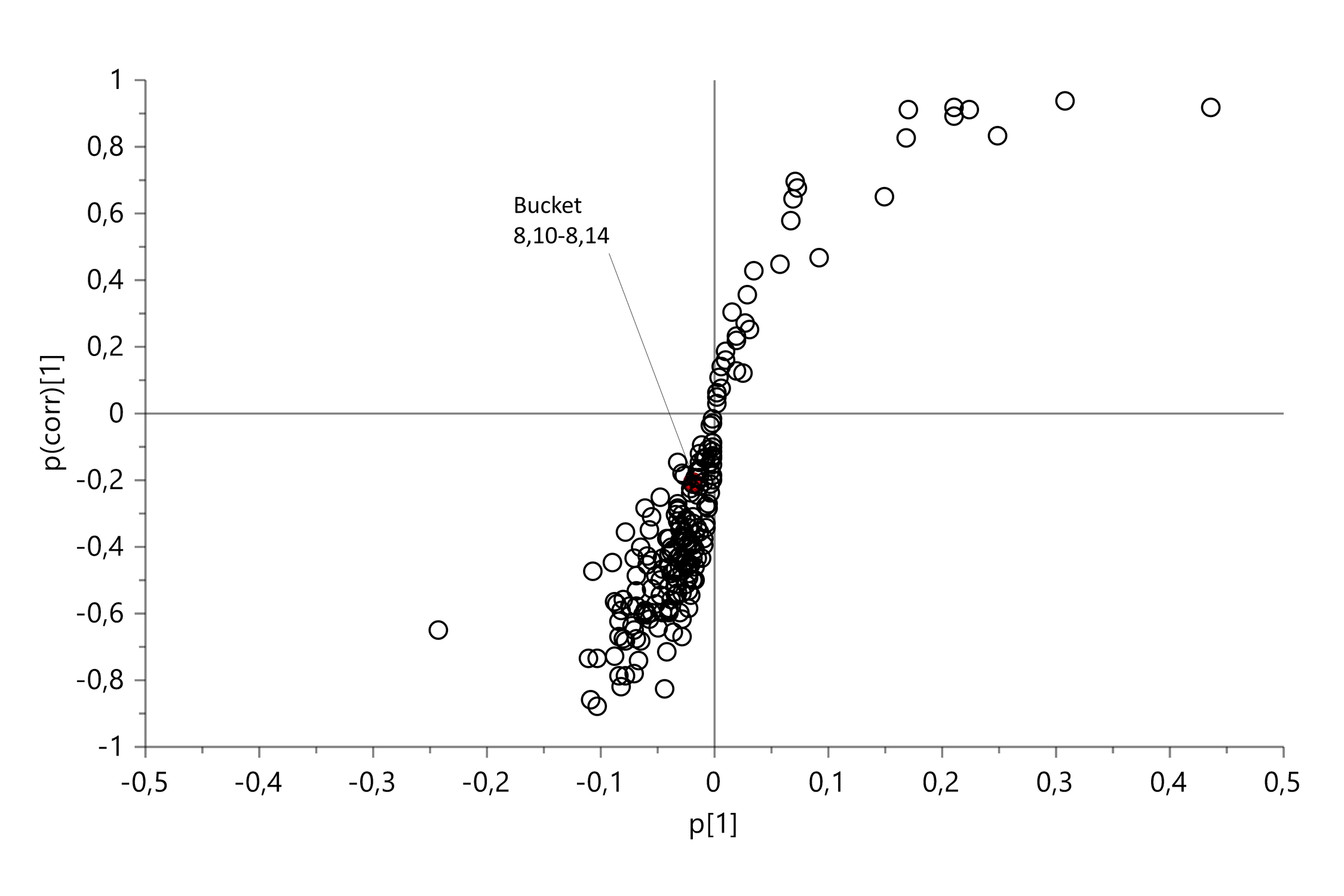

Supplement: S2 Fig — (TIF) [file pone.0266777.s002.tif]
